# A Cytochrome P450 3A4 Biosensor Based on Generation 4.0 PAMAM Dendrimers for the Detection of Caffeine

**DOI:** 10.3390/bios6030044

**Published:** 2016-08-18

**Authors:** Michael Müller, Neha Agarwal, Jungtae Kim

**Affiliations:** KIST Europe Forschungsgesellschaft mbH, Universität d. Saarlandes, Campus Geb. E7.1, Saarbrücken 66123, Germany; n.agarwal@kist-europe.de (N.A.); tais@kist-europe.de (J.K.)

**Keywords:** biosensor, cytochrome P450, electrochemistry, PAMAM dendrimers

## Abstract

Cytochromes P450 (CYP, P450) are a large family of heme-active-site proteins involved in many catalytic processes, including steroidogenesis. In humans, four primary enzymes are involved in the metabolism of almost all xenobiotics. Among these enzymes, CYP3A4 is responsible for the inactivation of the majority of used drugs which makes this enzyme an interesting target for many fields of research, especially pharmaceutical research. Since the late 1970s, attempts have been made to construct and develop electrochemical sensors for the determination of substrates. This paper is concerned with the establishment of such a CYP3A4-containing biosensor. The sensor was constructed by adsorption of alternating layers of sub-nanometer gold particle-modified PAMAM (poly-amido-amine) dendrimers of generation 4.0, along with the enzyme by a layer-by-layer assembly technique. Atomic force microscopy (AFM), quartz crystal microbalance (QCM), and Fourier-transformed infrared spectroscopy (FTIR) were employed to elucidate the sensor assembly. Additionally, the biosensor was tested by cyclic voltammetry using caffeine as a substrate.

## 1. Introduction

Cytochrome P450s (P450s) are a large superfamily of heme-thiolate enzyme proteins that can be found in all phyla. They generally catalyze formal oxidations, but reductions and rearrangements are also known. While they are mainly involved in many different catalytic processes, such as steroid-genesis [1] or the synthesis of antibiotics [2] in microorganisms, in humans they are also responsible for the metabolism of the majority of drugs and other exogenous substances [3,4]. Four main P450s (CYP3A4/5, CYP2D6, CYP2C8/9, and CYP1A2) are involved in these processes. P450s fulfill their energy demand by transferring equivalents via an electron transfer chain from co-factor Nicotinamide adenine dinucleotide phosphate (NADPH) along a FAD/FMN-containing P450 reductase to the heme active center of the P450s [5]. During substrate conversion, electrons are consumed to change the oxidation state of the heme central iron (Fe^II^/Fe^III^) to mediate the following conversion:

RH + O_2_ + NADPH + H^+^ → ROH + H_2_O + NADP^+^

Although all of the aforementioned human P450s are involved in xenobiotics metabolism, the majority of pharmaceuticals are metabolized by one enzyme, CYP3A4, including such diverse molecules as the anticonvulsant carbamazepine [6], the opioid analgesic codeine [7], the antibiotic erythromycin [8], the selective serotonin-reuptake inhibitor fluoxetin [9], and the anti-inflammatory drug hydrocortisone . Due to their involvement in, and the ability for, the catalysis of such a wide variety of compounds, considerable attention has been paid to these enzymes, especially from the pharmaceutical industry [10]. Understanding the roles of P450s in drug metabolism and their behavior in vivo is a key factor in the solution for problems like bioavailability, drug-drug interactions, and toxicity [11,12]. Increasing efforts in research are dedicated to the establishment and adaptation of P450s to use as biocatalysts for the synthesis of pharmaceuticals, like new antibiotics [13], and otherwise hard to obtain compounds, like leukotoxin B [14] or taxol [15]. In addition to this, P450s are gaining more interest as targets for drugs by themselves. CYP19 can be mentioned, a mammalian aromatase responsible for the transformation of androgens to oestrogens, which can be used as a therapeutic target for the treatment of hormone-responsive cancers [16]. Often testing of the P450s is based on assays, including complete cells or tissue preparations, which consumes time and is considered a bottleneck during the research process. This makes the establishment of alternative methods necessary and P450-modified electrodes offer many advantages, such as quick and simple measurements. Since the pioneering work of Scheller et al. [17] and Renneberg et al. [18], more and more effort has been put into the establishment of such biosensors, although, so far, no commercial product has arisen.

Adsorption of proteins directly to an electrode often results in denaturation of the biomaterial through the intrinsic instability of such enzymes [19], although there are a few reports of useable biosensors [20,21]. The key parameter for a successful enzymatic biosensor assembly is the connection between the transducer and enzyme, which is most often realized by using a special layer or film between both entities to keep the enzyme in a native state [22,23,24]. P450 biosensors have employed different compounds for the creation of such a layer, including conductive polymers [25], membrane-like structures from surfactants or insoluble lipids [26], proteins on thiol self-assembled monolayers [27], and many more [28,29]. They can be employed to sense substrates in a concentration-dependent manner in the range of low-micromolar to nanomolar detection with an electron transfer rate k_s_ between two and several hundred per second and limits of detection (LODs) ranging from low nanomol to micromole limits [30]. Alonso-Lumilo et al. incorporated CYP2B4 into a film of polypyrrole by electropolymerization of both compounds onto a gold electrode [25]. By embedding the protein into a polymer film they assumed to keep the enzyme in a more native state, as well as to reach a more favorable orientation to the electrode. By using chronoamperometry they could find a LOD of 0.289 µmol/dm^3^ and standard deviations for reproducibility and repeatability were 13.68% and 5.51%, respectively. In this regard, two publications by Udit et al. are worth mentioning as both of them describe a P450 electrode where the enzyme is embedded into a film, not of conducting polymers, but transfectants sodium-dodecyl-sulfate (SDS) [31] and didodecyldimethylammonium bromide (DDAB) [26], to exploit the before-mentioned advantages in combination with the *Bacillus megaterium* oxidase P450 BM3 and carbon electrodes. In both cases they could find a large influence of the surfactant material on the redox properties of the observed heme. Additionally, several reports of CYP3A4-containing biosensors can be found. Based on glassy carbon and a composite material from Nafion and cobalt (III) sepulchrate, two electron mediators, Hendricks et al. established such a biosensor for the detection of 2,4-dichlorophenol. They found a considerably low LOD of 0.043 g/L and a standard deviation of reproducibility of only 5.2%. Another CYP3A4 biosensor was realized by Rusling, based on a layer-by-layer assembly of alternating enzyme/polycation layers atop a 3-mercapto-propane-1-sulfonate (MPS)-modified gold electrode by electrostatic adsorption [32]. They could find Michaelis-Menten constants k_M_, depending on the used substrate, ranging from 271 to 1082 µM. Surely the most prolific group of this field is the one of Victoria Shumyantseva, which published a multitude of papers regarding P450 biosensors [33,34], including several concerned with CYP3A4. They found a high performance of electrode systems containing carbon electrodes and the synthetic membrane material DDAB doped by gold particles with k_M_ values, dependent on the used substrates benzphetamin, cholesterol, and lanosterol, of 13 µM, 830 µM, and 30 µM, respectively. Recently, this group started to include vitamins, like vitamin C, with known antioxidative properties to their biosensor setup, which stimulated the dose-dependent growth of the cathodic peak currents [35].

To increase the amount of immobilized, electroactive protein and, thus, the biosensor performance, more sophisticated strategies have been adopted: Krishnan et al., for instance, incorporated a fusion protein from P450 3A4 and its respective reductase to increase electroactivity and make the electron transfer more reliable by mimicking natural pathways. Such strategies have been adopted by several other groups who either fused or co-immobilized P450s with their respective reductases [36].

Another promising method to build an enzyme biosensor is the incorporation of dendrimers as such a “connective” layer. Dendrimers are a new class of polymers and are defined by their repetitively branched structure around a central core molecule. Their shape is typically symmetric around a multivalent core, and the molecules show a spherical, three-dimensional morphology that is ordered in generations [37]. While dendrimers with a low generation number exhibit an open structure, higher generation numbers result in a dense core-shell structure [38], which is why they are often used as drug or gene delivery vehicles [39,40,41]. This core-shell structure can be utilized to efficiently store active drug compounds inside of dendrimer molecules, not unlike packaging of drugs into nanoparticles [42]. Other applications of dendrimers include sensor compounds for ELISA [43], field effect transistors [44], impedimetric sensors [45], and their use in the generation of noble metal nanoparticles, especially gold particles [46].

Poly-amido-amine (PAMAM) dendrimers, which were the first to be commercialized (Starburst), are made from an ethylenediamine core with repeating sub-units of amide and amine functionality to further synthesize the molecule. Since a publication by Yoon et al. in 2000, few reports have included these PAMAM dendrimers as building blocks for construction of redox-active, heme enzyme biosensors with glucose oxidase, horseradish peroxidase, or myoglobin, where an assembly of several alternating protein/dendrimer layers was used [47].

Full-generation-number PAMAMs with amine functional surface groups are well-suited for the immobilization of P450s. On the proximal side to the active center, close to the heme, several chargeable amino acids function as docking sites for the P450 reductases and cytochrome B5 in vivo and mediate the bonding process next to hydrophobic and hydrogen bonding [48,49]. As charged moieties are usually rather evenly distributed across the surface of the enzyme it is suggested that such a patch with higher charge density will preferably bind to the PAMAM surface groups, leading to a more oriented immobilization of the enzyme. As native transfer pathways are followed this way, a higher number of electroactive P450 molecules can be expected.

We present a cytochrome P450 3A4 biosensor that includes PAMAM dendrimers of generation 4.0 as a connective layer between a gold electrode and the enzyme layer. These molecules were modified to include small to sub-nanometer sized gold particles inside their cavities as it is suggested to increase conductivity throughout the dendrimer layer. Furthermore, there are reports that nanoparticles are able to penetrate the usually insulating protein shell, thus aiding electron transfer to the enzyme’s active center. To construct the biosensor, a layer-by-layer assembly method (LBL assembly) was used in which the affinity between two oppositely-charged moieties and the resulting quasi-ionic bonding resulted in a structure of stacked monolayers. As gold surfaces are rather inert to many compounds, a negatively-charged layer of MPS was introduced, atop which the other layers where built. Furthermore, the generation 4.0 PAMAM dendrimers were modified with low- to sub-nanometer-sized gold particles to increase electron transfer through the dendrimer layer [50].

After assembly of the sensor was established, it was used for the amperometric sensing of the CYP3A4 substrate caffeine. Metabolism of caffeine in humans is a complex, multi-step process including different P450s (CYP1A2, CYP2C8/9, and CYP3A4), with CYP1A2 as the predominant one, which mediates either 1-, 3- or 7-N-demethylation or a C-8-hydroxylation [51]. To a lesser extent, CYP3A4 is involved in 7-N-demethylation or C-8-hydroxylation of the compound, resulting in theophylline or 1,3,7-trimethyluric acid, respectively. This makes caffeine an interesting compound to investigate the complex interplay between several human P450s and their substrates [52], and establishment of such a P450 biosensor for this field of research can help the progression thereof.

The detection method for the sensing of caffeine was cyclic voltammetry (CV). A typical electrochemical three-electrode setup made from a 30 mm × 20 mm platinum counter electrode, a silver/silverchloride reference electrode and the gold-based biosensor as the working electrode was used. With this setup, caffeine solutions in the range of 0.5 to 100 µM were tested.

## 2. Material and Methods

### 2.1. Materials

Purified human cytochrome P450 3A4 was used from Cypex Limited (Dundee, Scotland, UK ). All other chemicals were bought at highest available purity from Sigma Aldrich Chemie GmbH (Darmstadt, Germany) except the following: potassium nitrate, used as an electrolyte in the electrochemical experiments was from Merck KGaA (Darmstadt, Germany), trisodiumcitrate dihydrate and sodium dihydrogen phosphate were obtained from Carl Roth GmbH and Co KG (Karlsruhe, Germany); and the PAMAM G4.0 dendrimers were obtained via Sigma Aldrich Chemie GmbH from Dendritech Inc. (St. Louis, MO, USA).

A 100 mM phosphate buffer has been prepared from sodium-dihydrogen-phosphate and disodium-hydrogen-phosphate in appropriate ratios, and was used in all experiments. pH values were adjusted to suitable values with HCl or NaOH. For electrochemical experiments, 100 mM KNO_3_ was added to a pH 7.4 phosphate buffer as an electrolyte.

All experiments were conducted at room temperature (23 °C) unless otherwise stated.

An Autolab PGstat12 (Ecochemie, Utrecht, Netherlands) was used for all electrochemical experiments, UV-VIS measurements were conducted with using a Genesys 10s (Thermo Scientific Inc., Waltham, Massachusetts, USA). Fluorescence measurements were conducted with a Perkin Elmer LS45 (Waltham, Massachusetts, USA). A Q-Sense E1 was used for all QCM experiments. Fourier-transformed infrared spectroscopy (FTIR) was performed with a Bruker Vector 22 (Billerica, MA, USA) equipped with a Bruker A518 grazing angle unit with a fixed 80° angle. Atomic force microscopy was performed with a Park Systems NX10 AFM (Suwon, South Korea) equipped with a non-contact mode probe (PPP-NCLR-10, Nanosensors(Nauchatel, Switzerland)).

### 2.2. Methods

#### 2.2.1. Preparation of PAMAM G4.0-Au Nanocomposites

To increase conductivity of the PAMAM monolayers in the biosensor assembly, gold nanoparticles (Au NPs) were synthesized using citrate as a reducing agent, as first described by Turkevich et al. and expanded by Frens et al. [53,54]. This method was further modified to incorporate the amine-terminated PAMAM dendrimers of generation 4 to form Au/PAMAM composites. During this process, two differently-sized particle types were expected to form, depending on the interaction with either the primary or tertiary amine/amide moieties of the PAMAM molecule.

A molar ratio of Au^III^/PAMAM G4 of 10:1 was used by thoroughly mixing 1 mL of a 1 mM solution of PAMAM G4 dendrimers and 1 mL of a 10 mM solution of HAuCl_4_ for 30 min by magnetic stirring. Subsequently, the mixture was heated to 96 °C, and 0.5 mL of a 100 mM trisodiumcitrate dihydrate solution was added. This mixture was kept at 96 °C under constant stirring for another 3 h to ensure complete reduction of Au^III^ to Au°. After incubation, the solution was centrifuged at 21,000 rpm for 60 min to remove the larger particles from the solution keeping only the sub- to low-nanometer-sized gold particles. After centrifugation, the slightly-colored supernatant was carefully removed and stored in new 1.5 mL tubes.

Before usage of the PAMAM G4.0-Au nanocomposites, UV-VIS and fluorescence measurements were performed to characterize the freshly constituted dendrimer/Au NP solutions. After obtaining these nanocomposites and characterizing them, they were used without further modification for the assembly of the complete CYP3A4 biosensor.

#### 2.2.2. Layer-by-Layer Assembly of the Au/MPS/G4.0-Au/CYP3A4 Biosensor

The CYP3A4-containing biosensor was assembled layer-by-layer (LBL). The individual layers were adsorbed in a sequential way through electrostatic attraction of oppositely charged moieties on the molecules’ surface. By adjusting the pH of the buffer solutions to values either below or above the isoelectric point (pI) of the respective compound, charge can be introduced to the molecule, either by protonation (i.e., NH_2_) or deprotonation (i.e., OH) [55].

For the preparation of the biosensor, either 10 × 20 mm gold-coated glass slides or gold-coated 1.4 cm^2^ AT-cut quartz crystals (LOT QD) were used as a substrate. Adsorption of the dendrimer-gold composite and CYP3A4 was quantitatively monitored in real-time by a quartz crystal microbalance (QCM, Q-Sense E1, LOT QD).

The Au slides were first immersed in a volume of isopropanol, sonicated for at least 15 min and dried by N_2_ stream. The slides were cleaned in a 5:1:1 mixture of ddH_2_O:NH_3_ (28%–32%):H_2_O_2_ (30%) for 15 min at 75 °C to remove organic compounds through oxidation, rinsed extensively with ddH_2_O and ethanol and then dried again by N_2_ stream. To introduce a negative charge to the Au surface, the electrodes were left in a 5 mM pH 5.6 solution of sodium-3-mercapto-1-propane-sulfonate (Na-MPS in ddH_2_O) for 24 h to form a monolayer through Au-S covalent bonding. After rinsing with ddH_2_O and ethanol and subsequent N_2_ stream drying, a 400 µM PAMAM G4.0-Au sample in a pH 5.0 phosphate buffer was drop-cast to cover the whole Au/MPS electrode and was incubated for 3 h to ensure complete coverage of the MPS surface with dendrimer molecules. Since generation 4 PAMAM dendrimers possess a pI of 9.3, deviation of the pH below this value causes protonation of the primary amine groups so a positive charge can be introduced to the dendrimer surface and, thus, enable binding to the negatively-charged sulfonate groups of the immobilized MPS molecules. After again rinsing with ddH_2_O and ethanol and N_2_ stream drying, the Au/MPS/PAMAM G4-Au sample was further processed through adsorption of the CYP3A4 (1 mM in phosphate buffer, pH 7.4) for another 3 h and was rinsed as before.

During all stages of the LBL assembly, samples were taken to be characterized by AFM and FTIR spectroscopy, and samples were prepared to monitor adsorption of the PAMAM G4.0-Au and CYP3A4 by quartz crystal microbalance. To obtain AFM pictures, several 2 and 5 µm^2^ areas were scanned with a non-contact-mode probe, and the surface morphologies were compared, as was the surface roughness R_q_, which was calculated by using an analysis option of the AFM software. FTIR spectroscopy was performed for the qualitative identification of the different biosensor layers between 4500 and 450 cm^−1^ by using an 80° grazing-angle module.

#### 2.2.3. Electrochemical Experiments using Au/MPS/PAMAM G4.0-Au/CYP3A4 Biosensor

A general three-electrode cell setup was used for the electrochemical measurements with a 30 mm × 20 mm Pt counter electrode and an Ag/AgCl reference electrode. The 20 mm × 10 mm gold electrode with the biosensor assembly as reported earlier was used as the working electrode. Electrochemical experiments were carried out at room temperature (25 °C) and in a 50 mM, pH 7.4 phosphate buffer containing 50 mM potassium nitrate. All measurements were carried out in aerobic conditions. The data were recorded with an Autolab PGSTAT12 and GPES software.

A first set of CV experiments were conducted using a 10 mM solution of potassium ferricyanide at each assembly stage of the biosensor to measure changes in conductivity among the individual layers.

A second set of experiments was conducted using caffeine as substrate for CYP3A4 at a concentration range of 0.5 to 100 µM to probe the sensor response. CVs using buffer only were also conducted, as well as CVs before and after incubation for 30 min with CYP3A4 inhibitor erythromycin (10 mM) [8].

## 3. Results and Discussion

### 3.1. Preparation of PAMAM G4.0-Au Nanocomposites

To increase the conductivity of the PAMAM G4 layer, Au nanoparticles were synthesized in the presence of the dendrimers by the citrate method. While low-generation PAMAM dendrimers exhibit a rather open structure, which results in large Au NPs formed through interaction with the inside of the molecule, higher-generation PAMAM dendrimers possess a core-shell structure that restricts the size of the formed particles to that of several Au atoms, depending on the generation. Approximately two populations of particles are formed when using G4.0 PAMAM dendrimers. Only the size-constrained ones are necessary to enhance the electrical conditions of the dendrimers.

After mixing of the generation 4.0 PAMAM dendrimers with the gold-tetrachlorate, an immediate color change from yellow to orange was observed which was caused by complexation of the AuCl_4_^−^ ions with primary and tertiary amines of the PAMAM molecule.

After production and separation of the Au NPs, the particles were characterized by UV-VIS and fluorescence spectroscopy. While the large-nanometer-sized Au particles (>2 nm) could be characterized by an absorption peak from the plasmonic vibration at approximately 520 nm, Au particles below 1.2 nm in size exhibited a large increase in fluorescence intensity caused by a simplified energy bend structure [56].

**Figure 1:**
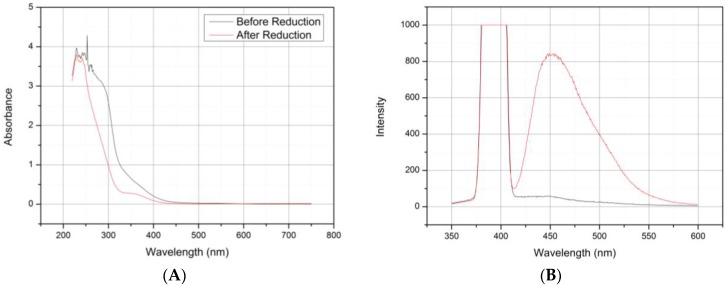
(**A**) UV-Vis scans of the Au/PAMAM G4.0 solution before and after reduction of gold by citrate. (**B**) Fluorescence scans with excitation at 384 nm of the supernatant (red) and resuspended pellet (black) after centrifugation.

UV-VIS scans were performed in the range of 250 to 650 nm, first, to check for removal of the >2 nm particles, which usually show a characteristic absorption peak at approximately 520 nm and, second, to show the formation of smaller particles inside the dendrimer’s cavities. These low- to sub-nanometer particles could be identified by a low-level absorption peak between 350 and 400 nm, corresponding to the fluorescence excitation wavelength of the Au particles [56]. As seen in Figure 1A, no absorbance peak was seen between 500 and 550 nm which indicates the efficient removal of these particles through centrifugation. Another absorption peak was observed at 364 nm, which is in good correlation with the literature data. This peak value was used in subsequent fluorescence experiments as the excitation wavelength. Figure 1B shows two scans from 350 to 600 nm, one of the supernatant, which contained the PAMAM G4-Au nanocomposites, and one from the resuspended “pellet”, formed through centrifugation and containing the large-nanometer Au particles. Both samples were excited at 384 nm, but only the supernatant sample shows an emission signal at 451 nm. The one containing the large particles showed no visible signal at this position because of the more complex bend structure in the particles containing a high number of gold atoms. 

It was concluded that both analysis methods are sufficient to validate the small particle formation inside of the dendrimers, as only these show fluorescence signals. Furthermore, no plasmonic absorption from gold particles could be found in UV-VIS scans in the range from 500 to 550 nm, which suggests that few to no larger Au particles were left over that could interfere with the sensor assembly.

### 3.2. Layer-by-Layer Assembly of the Au/MPS/G4.0-Au/CYP3A4 Biosensor

With the PAMAM G4.0-Au nanocomposites, CYP3A4 biosensors were constructed. LBL assembly of the biosensor was observed by QCM for quantitative information, by AFM for visual observation, and by grazing-angle FTIR spectroscopy for the detection of monolayers for qualitative information.

QCM experiments to adsorb the PAMAM-Au nanocomposites to MPS were conducted using the standard flow module of the Q-Sense device at a flow rate of 100 µL/min. The frequency change of the 13th overtone, which is the 13th harmonic oscillation of the resonance frequency, was monitored and subsequently used for the calculation of mass increase.

**Figure 2:**
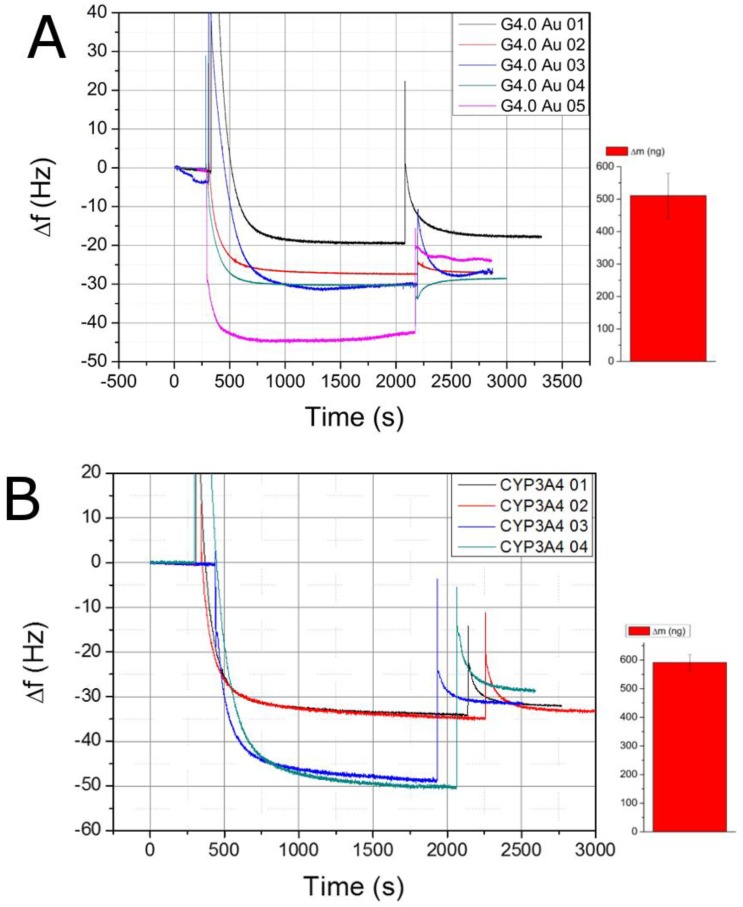
Adsorption process of PAMAM G4.0-Au to Au/MPS electrode at pH 5.0 (**A**) and CYP3A4 adsorption to Au/MPS/G4.0-Au at pH 7.4. (**B**) After obtaining a stable frequency response with phosphate buffer the sample was introduced and after completion of the adsorption process a washing step was conducted to remove only deposited or non-specifically bound moieties. These recorded ∆f values were used for the calculation of ∆m by Q-Tools software.

**Figure 3:**
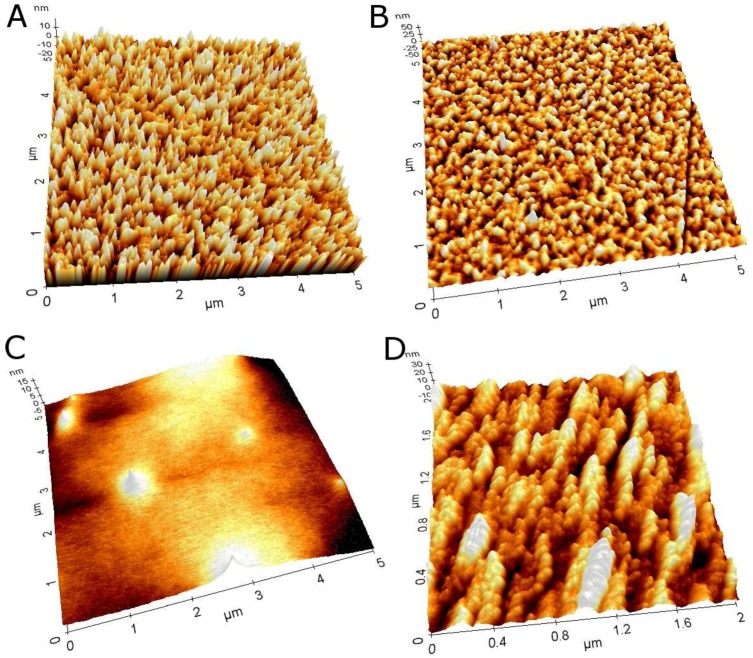
Atomic force microscope images of 5 × 5 µm (2 × 2 µm for CYP3A4 layer) areas of (**A**) Au, (**B**) Au/MPS, (**C**) Au/MPS/PAMAM G4.0-Au and (**D**) Au/MPS/PAMAM G4.0-Au/CYP3A4 respectively. No significant difference can be seen between (**A**) and (**B**) which can be explained by size considerations of the MPS compounds. In C, a change in surface morphology can be seen and is in good agreement with literature data on PAMAM dendrimer adsorption to gold while protein adsorption increases surface roughness again.

Figure 2A shows different experiments to create a PAMAM G4.0-Au monolayer on top of Au/MPS. Four of the five experiments showed a narrow frequency range. This can be attributed to the nature of the electrostatic bonding, which generally avoids PAMAM/PAMAM binding due to similar charge and to complete monolayer formation. Compared to adsorption experiments with the pure G4.0 PAMAM compound, a mass increase of ~78 ng (510.74 ng) was observed (data not shown) that was related to the number of gold particles either located inside of the dendrimer molecule or from leftover large nanoparticles chemisorbed to the MPS surface. AFM images (Figure 3C) from the Au/MPS/PAMAM G4.0-Au assembly show the typical smooth morphology changes after adsorption of the PAMAM dendrimers (R_q_ from 6.2 to 1.4 nm).

**Figure 4:**
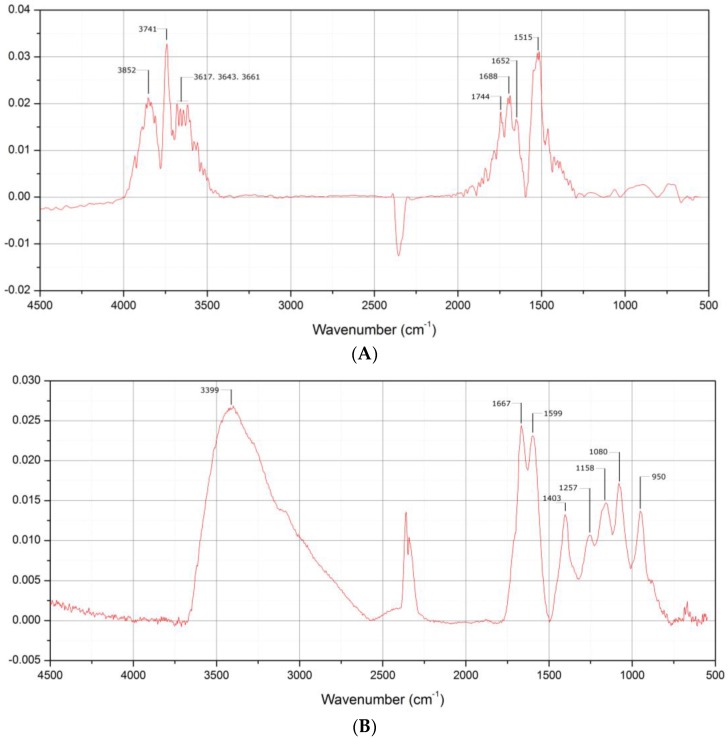
Grazing angle FTIR spectrograms of Au/MPS (**A**) and Au/MPS/PAMAM G4.0-Au (**B**). While in A only non-specific peaks appeared which could not be attributed to the MPS molecule functional groups like S-H or C-S (**A**), Intense absorption bends could be observed from the amine stretching vibration at 3399 which is a cumulative one from primary surface and tertiary amines and amides. Also, absorption could be observed for amine bending vibrations at 1667 and 1599 which is in good agreement to literature data and enough for identification of the compound.

Grazing-angle FTIR spectroscopy was performed at different biosensor assembly stages, and scans were obtained in the range of 4500 to 500/cm. Unfortunately, the base layer compound MPS was very small, so formation of the MPS layer could not be observed, as signals were below the detection limit of the spectrometer (Figure 4A). Comparison to Au/MPS/PAMAM G4.0-Au (Figure 4B) showed a huge absorption peak at 3399/cm that was attributed to primary and tertiary amine stretching vibration. Its magnitude can be explained by the high number of these functional groups. Corresponding to that peak, a typical double peak was observed at 1667 and 1599/cm, attributed to the amine’s bending vibration. Furthermore, C=O bending vibration was observed at 1403, but there was only a slight shoulder of the stretch mode at 1703/cm originated from the amide groups of the dendrimer branches.

After establishment of the PAMAM layer formation, experiments were conducted for the adsorption of the CYP3A4 to the dendrimer layers. Since enzymes are usually highly complex compounds with many different functional groups, charge can be easily introduced to the molecules, so adsorption experiments were conducted at neutral pH (7.4) to avoid possible protein denaturation at more extreme values. QCM data yielded a higher variability (Figure 2B) than expected which we attributed to protein-protein non-specific bonding or the appearance of protein dimers, which might have been increased due to the nature of the protein, which possessed a homologous membrane-binding region. The mean ∆m for these experiments was 591.229 ± 27.739 ng which is 52.9 ng higher than the suggested theoretical value. FTIR scans were conducted to identify the CYP3A4, but the resulting peak patterns were too complex to interpret.

The complete CYP3A4 biosensors were subsequently stored at 4 °C until use for the concentration-based sensing of the substrate caffeine.

### 3.3. Au/MPS/PAMAM G4.0-Au/CYP3A4 Electrochemistry Using Potassium-Ferricyanide

Ferricyanide [Fe(CN)_6_]^3−^ consists of a Fe^3+^ center bound in octahedral geometry to six cyanide ligands. Its iron is in a low spin state and can be easily reduced to ferrocyanide [Fe(CN)_6_]^4−^, which is a ferrous (Fe^2+^) derivative. This compound is a standard test subject in electrochemistry as a marker because it shows nearly reversible redox behavior. Here it was used to measure the changes in conductivity among the different biosensor assembly stages.

**Figure 5:**
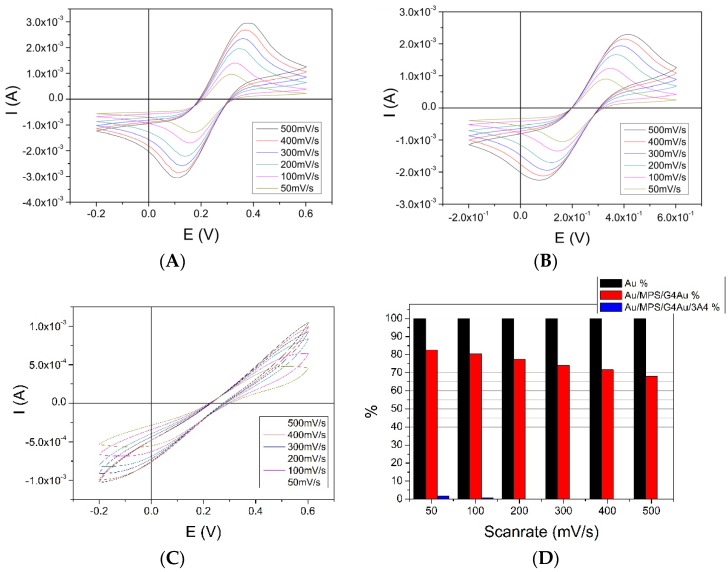
Cyclic voltammograms of 10mM Potassium Ferricyanide using different assembly stages of the CYP3A4 biosensor as working electrodes. The scan rate was varied between 50 and 500 mV/s. A Pt counter and an Ag/AgCl reference electrode were used. It can be seen that conductivity is reduced in all cases compared to the bare gold working electrode (**A**). Depending on the scan rate. Au-NP-doped PAMAM G4.0 (**B**) shows only reduction of conductivity between 83.97 (50 mV/s) to 68.72% (500 mV/s) while the biosensor shows no detectable current after adsorption of the CYP3A4 (**C**). (**D**) shows an overview of the collected data where the current response of the bare gold electrode was used as 100% value.

A 10 mM solution of ferricyanide (in phosphate buffer pH 7.4 plus 100 mM KNO_3_) was used with a 10 × 20 mm sensor, starting with bare gold and continuing with all biosensor layers. The obtained peak current data from the bare gold sample was defined as 100%. All experiments were conducted at varying scan rates in the range of −0.2 to 0.6 V. Comparing Figure 5A,C, peak reductive current I_P, C_ was reduced between 16.03% and 31.28%, depending on the scan rate, which is an increase compared to pure PAMAM G4.0 (64.81% to 48.39%) of more than 20% at a low scan rate. No detectable cathodic peaks could be identified after adsorption of the CYP3A4, as proteins are generally rather insulating entities. Additionally, in enzyme-based biosensor research, this effect is a prerequisite for a successful construction, as side reactions at the bare electrode material or one of the former layers generally have to be avoided. As the biosensor showed only a slight decrease in the peak current at low scanrates, 50 mV/s was chosen for further experiments.

### 3.4. Concentration-Based Sensing of the Au/MPS/PAMAM G4.0-Au/CYP3A4 Biosensor of Caffeine/Cyclic Voltammetry

**Figure 6:**
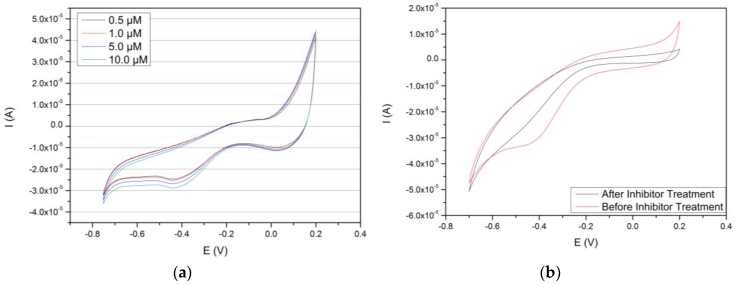
Cyclic voltammograms of the Au/MPS/PAMAM G4.0-Au/CYP3A4 electrode. (**a**) Shows the behaviour of the electrode with 0, 0.5, 1.0, 5.0 and 10.0 µM of caffeine, respectively. An increasing, reductive current can be observed at ~ −400 mV. (**b**) Depicts two voltammograms produced before and after treatment with CYP3A4 inhibitor eryhtromycin and under presence of substrate caffeine. An almost total disappearance of the typical P450-related reduction peak can be observed.

Cyclic voltammetry was performed at 50 mV/s with varying concentrations of caffeine. In the human body, caffeine is metabolized by several P450s, including CYP3A4. During the CYP3A4-mediated step, caffeine is catalyzed to theophylline, which is further processed to 1-methyluric acid, which is finally removed from the body. It is used as a model substrate because it has high affinity to CYP3A4 and is highly water soluble. As seen in Figure 6A, all samples containing caffeine showed more or less well-developed cathodic peaks at −406 ± 4 mV, which is in good agreement with literature data that show general heme reduction potentials between −380 and −420 mV [57]. Even at 0.5 µM of caffeine, a cathodic peak can still be observed, although only slightly. However, in experiments under anaerobic conditions without a substrate present, oxidative currents resulting from the heme Fe^II^/Fe^III^ redox couple could not be observed.

To determine the dependence of the obtained signal to the enzymatic substrate conversion, CVs were performed with a 10 µM solution of caffeine before and after incubation with a 10 mM erythromycin sample. As can be seen in Figure 6B cathodic signals completely disappears from occupying the active center with this high affinity substrate/inhibitor.

## 4. Conclusions

In this report, we presented the construction of a CYP3A4-containing biosensor using PAMAM dendrimers as a connective layer between a gold electrode and the enzyme. A LBL method was used for the construction of such a sensor, where monolayers were formed from single compounds through electrostatic bonding of charged moieties. The PAMAM G4.0 dendrimers were modified to increase their conductivity with small- to sub-nanometer-sized gold particles, which were formed inside of the dendrimer cavities through interaction with tertiary amine and amide moieties. The existence of these particles was demonstrated by UV-VIS and fluorescence measurements, as these gold particles showed a highly increased fluorescence intensity compared to larger particles through plasmonic properties. Assembly of the biosensor was established in a conclusive, repeatable manner using PAMAM G4.0-Au, which showed a well-established monolayer formation in AFM, QCM and qualitative FTIR measurements. Formation of the CYP3A4 layer was consistent, but adsorbed masses seemed to be slightly higher than calculated values, which probably resulted from non-specific protein-protein binding, although AFM images resembled rather a monolayer formation with oriented attachment of the protein, as shown by Ferrero and Andolfini et al. [58].

Cyclic voltammetry with only buffer under anaerobic conditions was performed to determine formal potential as well as electron transfer rate k_s_ via information obtained from the heme’s Fe^II^/Fe^III^ cycling. Unfortunately, the CYP3A4 biosensor did not yield any current response under these conditions, so calculation of these parameters was not possible and has to be further researched. A reason for the lack of these signals could be the either be the slow electron transfer kinetics to and from the electrode under the used conditions or signal intensities lie below the capacitive current detection limit. This problem must be addressed in future work as many other approaches for such a sensor yielded exactly this response.

Aerobic CVs showed well-defined reduction peaks at a potential of −406 ± 4 mV in the presence of caffeine from 0.5 µM to 10 µM. Peak signal intensities are in the range of nano amperes, which is consistent with other P450 biosensors [29], and can even compare to electrode assemblies containing P450/CPR fusion proteins [57] where signals were obtained in the 10^−7^ A range. So far, a LOD was established at 0.5 µM of caffeine as lower concentrations of this compound did not yield any measurable, significant current response. As not all reports on P450 biosensors concern themselves with the establishment of such parameters, only a few could be found with similar or lower detection limits; worth mentioning may be the polypyrrole-based CYP2B4 biosensor of Alonso-Lumilo et al. [25]. The origin of the obtained current signals could furthermore be attributed to the enzymatic reaction, as blocking its functionality with CYP3A4 inhibitor erythromycin resulted in the disappearance of the reductive current during cyclic voltammetry.

It has to be mentioned that no direct proof could be obtained from the set of experiments conducted. As both heme and oxygen reduction proceed simultaneously at the electrode surface, interpretation of the signal generation is considerably complicated and makes analysis of the formed product necessary by methods like mass spectrometry [59]. Large scale electrolysis coupled with a fitting analytical method comes to mind.Overall, this report showed the feasibility of using modified, generation 4 PAMAM dendrimers for the construction of an electrochemical CYP3A4 biosensor. Although other reports make use of several, alternating layers of dendrimer/protein to obtain a sufficiently large electrical signal [47], it appears that one alternating layer of dendrimer and protein can already yield a sufficient sensor response.
